# Retraction statement: High‐intensity focused ultrasound inhibits invasion and metastasis of colon cancer cells by enhancing microRNA‐124‐mediated suppression of STAT3

**DOI:** 10.1002/2211-5463.13507

**Published:** 2022-11-15

https://doi.org/10.1002/2211-5463.12642

The above article published online on 13 April 2019 in Wiley Online Library (wileyonlinelibrary.com), has been retracted by agreement between the Editor‐in‐Chief, John Wiley and Sons Ltd and the authors. The retraction has been agreed following an investigation based on allegations raised by a third party, which revealed that some images in this article are inappropriate duplications of images from previously published articles [1, 2, 3]. Thus, the editors consider the conclusions of this manuscript substantially compromised.
